# Supplementary material for: ATP and small amphiphilic molecules act as molecular matchmakers to fine-tune FET protein clusters
Source: Commun Chem. 2025 Nov 29;8:386. doi: 10.1038/s42004-025-01807-5 (PMC12673092; doi:10.1038/s42004-025-01807-5)
Supplement: Supplementary file 2 — Supplimentary Information [file 42004_2025_1807_MOESM2_ESM.pdf]

Supporting Information for

ATP and Small Amphiphilic Molecules Act as Molecular Matchmakers to Fine-Tune FET Protein Clusters

*Mrityunjoy Kar**

Leibniz-Institut für Polymerforschung Dresden e.V.,
Dresden, Germany

Materials

List of reagents, sources, and vendor identifiers, if any

REAGENTS	SOURCE	IDENTIFIER
**CHEMICALS**		
HEPES	Merck Germany	7365-45-9
Potassium chloride (KCl)	Merck Germany	7447-40-7
Glycerol	VWR chemicals	56-81-5
cOmplete^{TM}	Roche Germany	11697498001
Imidazole	Merck Germany	288-32-4
Dithiothreitol (DTT)	Alfa Aesar Germany	578-51-7
Maltose	Merck Germany	6363-53-7
Potassium hydroxide	Merck Germany	1310-58-3
Adenosine 5'-triphosphate (ATP) sodium salt	Jena Bioscience	987-65-5
Adenosine 5'-diphosphate (ADP) sodium salt	Merck Germany	20398-34-9
Adenosine 5'-monophosphate (AMP) sodium salt	Merck Germany	149022-20-8
Sodium tripolyphosphate (STPP)	Merck Germany	7758-29-4
Sodium pyrophosphate (SPP)	Merck Germany	13472-36-1
Sodium phosphate (SP)	Merck Germany	7601-54-9

Sodium toluene sulfonate (NaTS)	Merck Germany	657-84-1
Sodium xylene sulfonate (NaXS)	Merck Germany	1300-72-7
1,6 hexanediol (HD)	Merck Germany	629-11-8
Triethylene glycol	Merck Germany	112-27-6
Magnesium Acetate	Merck Germany	16674-78-5
**Bacterial and Virus Strains**		
Sf9 cells	Expression Systems	Cat#94-001F
**Recombinant proteins**		
FUS-SNAP	Kar et. al. 2022 [1]	TH0901
FUS	Kar et. al. 2022 [1]	TH0901
TAF15-SNAP	Kar et. al. 2022 [1]	TH1203
EWSR1-SNAP	Kar et. al. 2022 [1]	TH1276

Amino acid sequences of proteins used in studies

1. FUS-SNAP: This sequence includes full-length FUS (unshaded), a linker that is cleavable by a TEV protease (shaded in yellow), and the SNAP (shaded in gray).

MASNDYTQQATQSYGAYPTQPGQGYSQQSSQPYGQQSYSGYSQSTDTSGYGQSSYSSYGQSQ
NTGYGTQSTPQGYGSTGGYGSSQSSQSSYGQQSSYPGYGQQPAPSSTSGSYGSSSQSSSYGQ
PQSGSYSQQPSGGQQQSYGQQQSYNPPQGYGQQNQYNSSSGGGGGGGGGNYGQDQSSMSSG
GGSGGGYGNQDQSGGGGSGGYGQQASDRGGRGRGGSGGGGGGGGGYNRSSGGYEPRGRGGG
RGGRGGMGGSDRGGFNKFGGPRDQGSRHDSEQDNSDNNTIFVQGLGENVTIESVADYFKQIG
IIKTNKKTGQPMINLYTDRETGKLKGEATVSFDDPPSAKAAIDWFDGKEFSGNPIKVSFATR
RADFNRGGGNGRGGRGRGGPMGRGGYGGGGSGGGGRGGFPSGGGGGGGQQRAGDWKCPNPTC
ENMNFSWRNECNQCKAPKPDGPGGGPGGSHMGGNYGDDRRGGRGGYDRGGYRGRGGDRGGFR
GGRGGGDRGGFGPGKMDSRGEHRQDRRERPYGAPGSSSGRENLYFQGMDKDCEMKRTTLDSP
LGKLELSGCEQGLHRIIFLGKGTSAADAVEVPAPAAVLGGPEPLMQATAWLNAYFHQPEAIE
EFPVPALHHPVFQQESFTRQVLWKLLKVVKFGEVISYSHLAALAGNPAATAAVKTALSGNPV
PILIPCHRVVQGDLDVGGYEGGLAVKEWLLAHEGHRLGKPGLG

2. FUS

MASNDYTQQATQSYGAYPTQPGQGYSQQSSQPYGQQSYSGYSQSTDTSGYGQSSYSSYGQSQ
NTGYGTQSTPQGYGSTGGYGSSQSSQSSYGQQSSYPGYGQQPAPSSTSGSYGSSSQSSSYGQ
PQSGSYSQQPSYGGQQQSYGQQQSYNPPQGYGQQNQYNSSSGGGGGGGGGNYGQDQSSMSS
GGGSGGGYGNQDQSGGGGSGGYGQQASDRGGRGRGGSGGGGGGGGGYNRSSGGYEPRGRGG
GRGGRGGMGGSDRGGFNKFGGPRDQGSRHDSEQDNSDNNTIFVQGLGENVTIESVADYFKQI
GIIKTNKKTGQPMINLYTDRETGKLKGEATVSFDDPPSAKAAIDWFDGKEFSGNPIKVSFAT
RRADFNRGGGNGRGGRGRGGPMGRGGYGGGGSGGGGRGGFPSGGGGGGGQQRAGDWKCPNPT
CENMNFSWRNECNQCKAPKPDGPGGGPGGSHMGGNYGDDRRGGRGGYDRGGYRGRGGDRGGF
RGGRGGGDRGGFGPGKMDSRGEHRQDRRERPY

3. TAF15-SNAP: This sequence includes full-length Taf15 (unshaded), a linker that is cleavable by a TEV protease (shaded in yellow), and the SNAP tag (shaded in gray).

MSDSGSYGQSGGEQQSYSTYGNPGSQGYGQASQSYSGYGQTTDSSYGQNYSGYSSYGQSYSQ
SYGGYENQKQSSYSQQPYNNQGQQQNMESSGSQGGRAPSYDQPDYGQQDSYDQQSGYDQHQG
SYDEQSNYDQQHDSYSQNQQSYHSQRENYSHHTQDDRRDVSRYGEDNRGYGGSQGGGRGRGG
YDKDGRGPMTGSSGGDRGGFKNFGGHRDYGPRTDADSESDNSDNNTIFVQGLGEGVSTDQVG
EFFKQIGIIKTNKKTGKPMINLYTDKDTGKPKGEATVSFDDPPSAKAAIDWFDGKEFHGNII
KVSFATRRPEFMRGGGSGGGRRGRGGYRGRGGFQGRGGDPKSGDWVCPNPSCGNMNFARRNS
CNQCNEPRPEDSRPSGGDFRGRGYGGERGYRGRGGRGGDRGGYGGDRSGGGYGGDRSSGGGY
SGDRSGGGYGGDRSGGGYGGDRGGGYGGDRGGGYGGDRGGGYGGDRGGYGGDRGGGYGGDRG
GYGGDRGGYGGDRGGYGGDRGGYGGDRSRGGYGGDRGGGSGYGGDRSGGYGGDRSGGGYGGD
RGGGYGGDRGGYGGKMGGRNDYRNDQRNRPYGAPGSSSGRENLYFQGMDKDCEMKRTTLDSP
LGKLELSGCEQGLHRIIFLGKGTSAADAVEVPAPAAVLGGPEPLMQATAWLNAYFHQPEAIE
EFPVPALHHPVFQQESFTRQVLWKLLKVVKFGEVISYSHLAALAGNPAATAAVKTALSGNPV
PILIPCHRVVQGDLDVGGYEGGLAVKEWLLAHEGHRLGKPGLG

4. EWSR1-SNAP: This sequence includes full-length Ewsr1 (unshaded), a linker that is cleavable by a TEV protease (shaded in yellow), and the SNAP-tag (shaded in gray).

MASTDYSTYSQAAAQQGYSAYTAQPTQGYAQTTQAYGQQSYGTYGQPTDVSYTQAQTTATYG
QTAYATSYGQPPTGYTTPTAPQAYSQPVQGYGTGAYDTTTATVTTTQASYAAQSAYGTQPAY
PAYGQQPAATAPTRPQDGNKPTETSQPQSSTGGYNQPSLGYGQSNYSYPQVPGSYPMQPVTA
PPSYPPTSYSSTQPTSYDQSSYSQQNTYGQPSSYGQQSSYGQQSSYGQQPPTSYPPQTGSYS

QAPSQYSQQSSSYGQQSSFRQDHPSSMGVYGQESGGFSGPGENRSMSGPDNRGRGRGGFDRG
GMSRGGRGGGRGGMGSAGERGGFNKPGGPMDEGPDLDLGPPVDPDEDSDNSAIYVQGLNDSV
TLDDLADFFKQCGVVKMNKRTGQPMIHIYLDKETGKPKGDATVSYEDPPTAKAAVEWFDGKD
FQGSKLKVSLARKKPPMNSMRGGLPPREGRGMPPPLRGGPGGPGGPGGPMGRMGGRGGDRGG
FPPRGPRGSRGNPSGGGNVQHRAGDWQCPNPGCGNQNFAWRTECNQCKAPKPEGFLPPPFPP
PGGDRGRGGPGGMRGGRGGLMDRGGPGGMFRGGRGGDRGGFRGGRGMDRGGFGGGRRGGPGG
PPGPLMEQMGGRRGGRGGPGKMDKGEHRQERRDRPYGAPGSSSGRENLYFQGMDKDCEMKRT
TLDSPLGKLELSGCEQGLHRIIFLGKGTSAADAVEVPAPAAVLGGPEPLMQATAWLNAYFHQ
PEAIEEFPVPALHHPVFQQESFTRQVLWKLLKVVKFGEVISYSHLAALAGNPAATAAVKTAL
SGNPVPILIPCHRVVQGDLDVGGYEGGLAVKEWLLAHEGHRLGKPGLG

Supplementary table 1: Comparison of hydrodynamic diameter (d_h) measurements obtained from dynamic light scattering (DLS) and nanoparticle tracking analysis (NTA). DLS data includes the polydispersity index (PDI) of the mesoscale clusters, and NTA data includes the volume fractions of the clusters.

	DLS				NTA	
	Mean size (nm) @ 30 mins	PDI	Mean size (nm) @ 8 mins	PDI	Mean size (nm) @ ~8 mins	Volume fractions ($\phi_{cluster} \times 10^{-5}$)
0.25 μM FUS SNAP	281.74 ± 14.98	0.122 ± 0.048	244.32 ± 11.98	0.132 ± 0.052	225 ± 14.2	0.209 ± 0.033
0. 5 μM FUS SNAP	441.80 ± 74.15	0.119 ± 0.036	344.41 ± 35.88	0.125 ± 0.061	282 ± 20.5	0.448 ± 0.145
1 μM FUS SNAP	1,012.40 ± 72.95	0.172 ± 0.052	705.29 ± 107.69	0.157 ± 0.099	493 ± 17.32	2.407 ± 0.835
0.25 μM FUS SNAP with 1 mM ATP.Mg^{2+}	673.45 ± 104.06	0.167 ± 0.065	476.28 ± 31.73	0.114 ± 0.022	248.5 ± 10.96	0.305 ± 0.116
0.25 μM FUS SNAP with 2 mM ATP.Mg^{2+}	642.58 ± 70	0.127 ± 0.054	446.10 ± 30.72	0.137 ± 0.056	267.3 ± 16.19	0.392 ± 0.279
0.25 μM FUS SNAP with 5 mM ATP.Mg^{2+}	312.38 ± 45.93	0.146 ± 0.059	323.25 ± 60.26	0.153 ± 0.053	174 ± 12.85	0.126 ± 0.062
0.25 μM FUS SNAP with 10 mM ATP.Mg^{2+}	222.21 ± 69.29	0.318 ± 0.212	169.67 ± 30.62	0.279 ± 0.19	155.5 ± 11.84	0.0568 ± 0.044

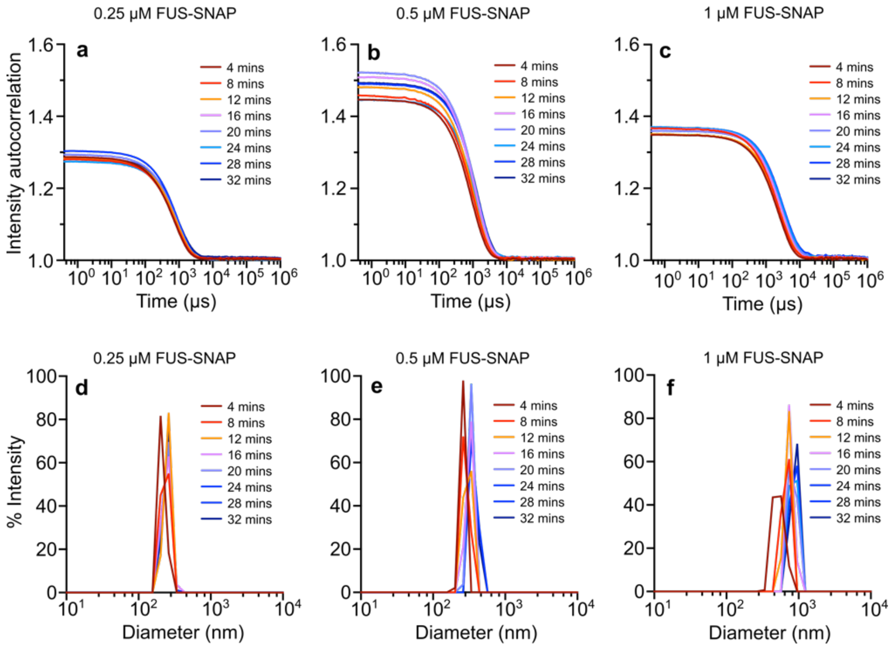

Supplementary figure 1: The autocorrelation function from DLS of solutions with varying concentrations of FUS-SNAP: 0.25 μM (a), 0.5 μM (b), and 1 μM (c) in 20 mM HEPES, pH 7.4, with 10 mM KCl. The size distributions of the scatterers are presented as % intensity derived from the corresponding intensity autocorrelation of DLS samples containing 0.25 μM (d), 0.5 μM (e), and 1 μM (f) FUS-SNAP.

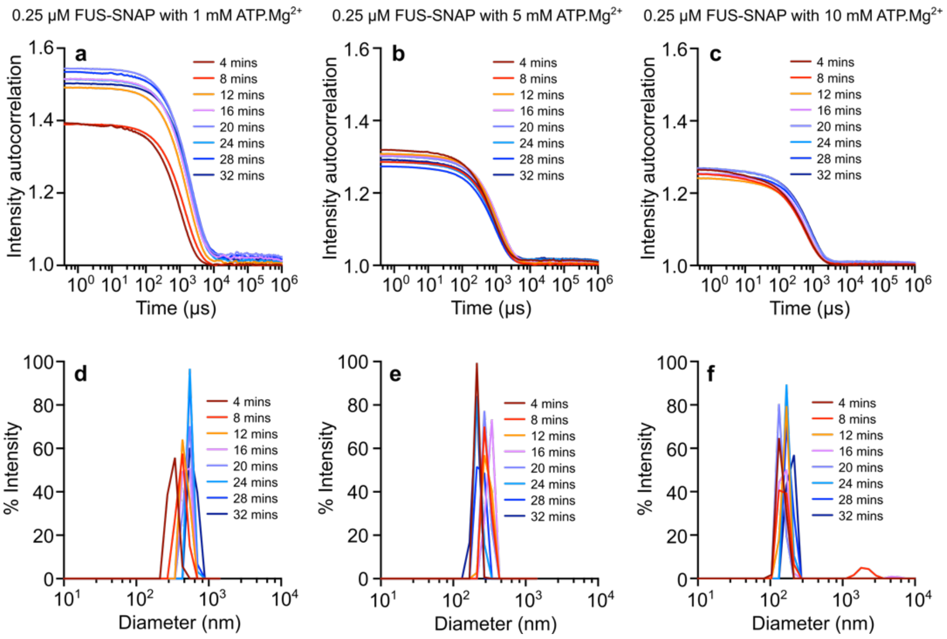

Supplementary figure 2: The autocorrelation function from DLS of solutions containing 0.25 μM FUS-SNAP with different concentrations of ATP.Mg^{2+}, 1 mM (a), 5 mM (b), and 10 mM (c) in 20 mM HEPES, pH 7.4, with 10 mM KCl. The size distributions of the scatterers are shown as percentage intensity derived from the intensity autocorrelation of DLS samples containing 1 mM (d), 5 mM (e), and 10 mM (f) ATP.Mg^{2+} with 0.25 μM FUS-SNAP.

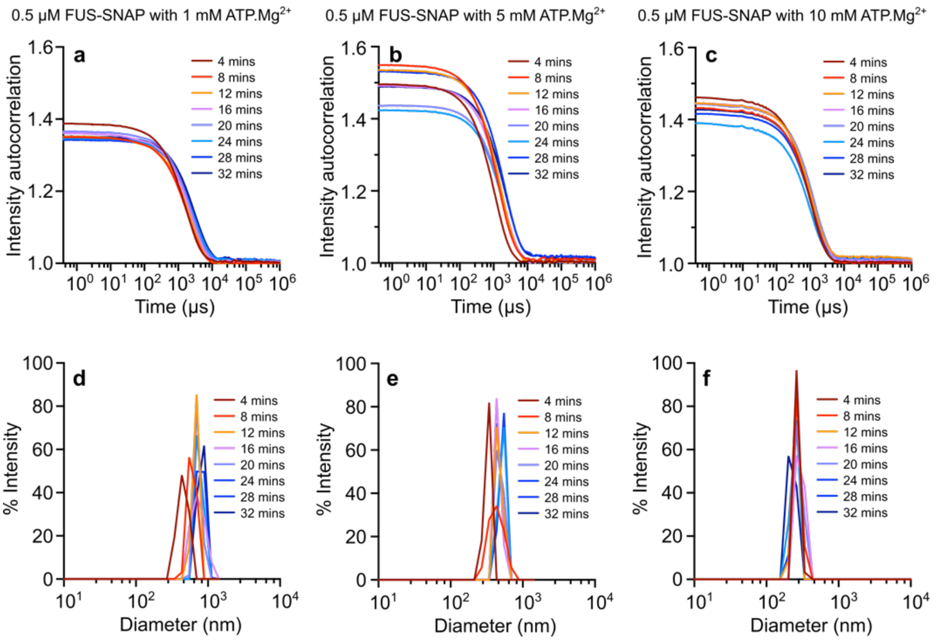

Supplementary figure 3: The autocorrelation function from DLS of solutions containing 0.5 μM FUS-SNAP with varying concentrations of ATP.Mg^{2+}, 1 mM (a), 5 mM (b), and 10 mM (c), in 20 mM HEPES, pH 7.4, with 10 mM KCl. The size distributions of the scatterers are presented as % intensity derived from the intensity autocorrelation of DLS samples containing 1 mM (d), 5 mM (e), and 10 mM (f) ATP.Mg^{2+} with 0.5 μM FUS-SNAP.

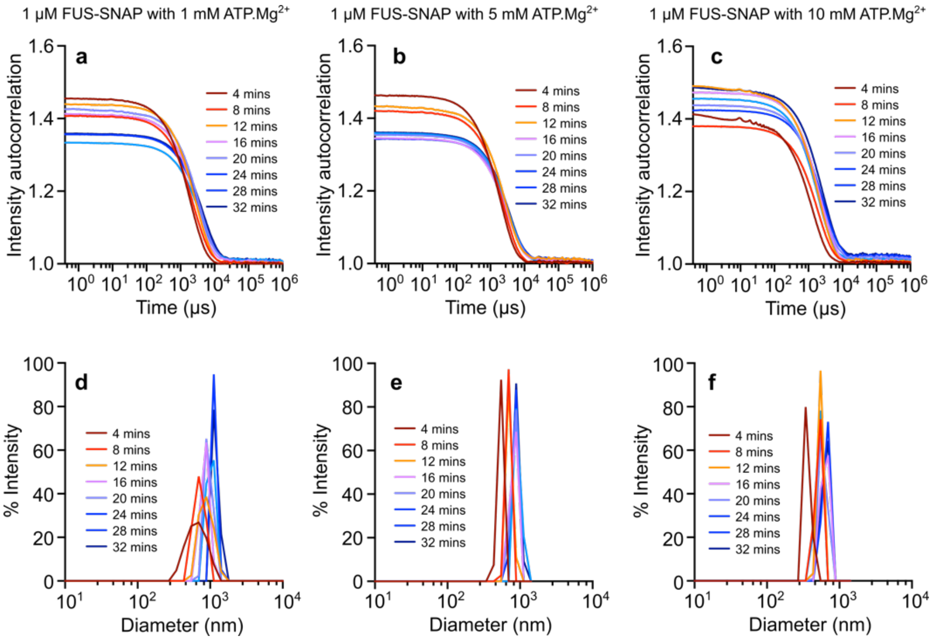

Supplementary figure 4: The autocorrelation function from DLS of solutions containing 1 μM FUS-SNAP with varying concentrations of ATP.Mg^{2+}: 1 mM (a), 5 mM (b), and 10 mM (c) in 20 mM HEPES, pH 7.4, with 10 mM KCl. The size distributions of the scatterers are shown as % intensity derived from the intensity autocorrelation of DLS samples containing 1 mM (d), 5 mM (e), and 10 mM (f) ATP.Mg^{2+} with 1 μM FUS-SNAP.

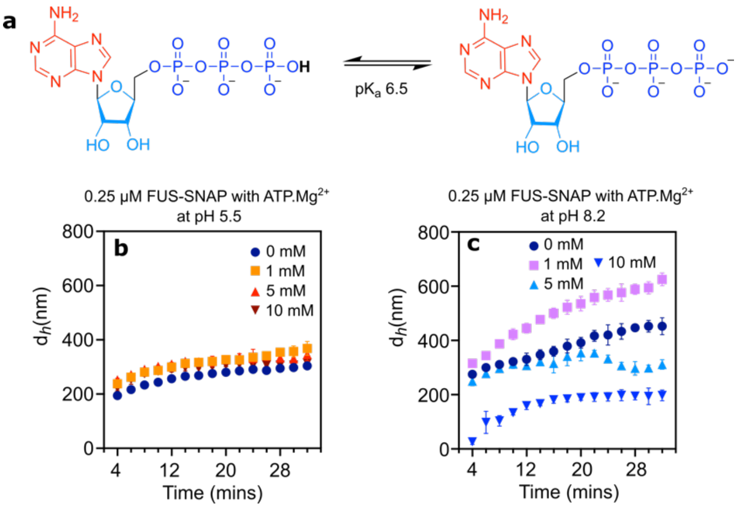

Supplementary figure 5: pH influences the formation of sub-saturation FUS-SNAP clusters with various concentrations of adenosine triphosphate (ATP.Mg^{2+}). Chemical structure drawing of ATP at pKa 6.5 (a). Dynamic Light Scattering data show the hydrodynamic diameter (d_h) of mesoscale clusters at 0.25 µM FUS-SNAP over 32 minutes with various concentrations of ATP at pH 5.5 (b), and pH 8.2 (c). Data are presented as mean ± SD, with n=3 independent samples.

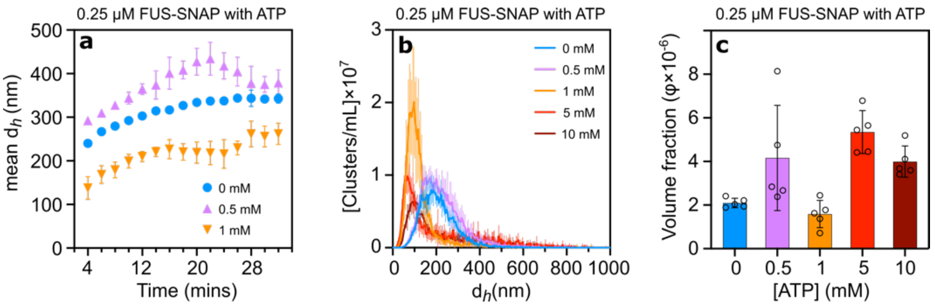

Supplementary figure 6: Adenosine triphosphate (ATP) without Mg^{2+} ion regulates the cluster size of sub-saturation FUS-SNAP clusters. Dynamic Light Scattering data show the hydrodynamic diameter (d_h) of mesoscale clusters at 0.25 µM FUS-SNAP over 32 minutes with various concentrations of ATP (a). NTA data for cluster hydrodynamic diameter at 0.25 µM FUS-SNAP with different ATP concentrations are shown in (b), while (c) displays the corresponding relative abundance of clusters as volume fraction. Data are presented as mean ± SD, with n=3 (DLS) and n=5 (NTA) independent samples.

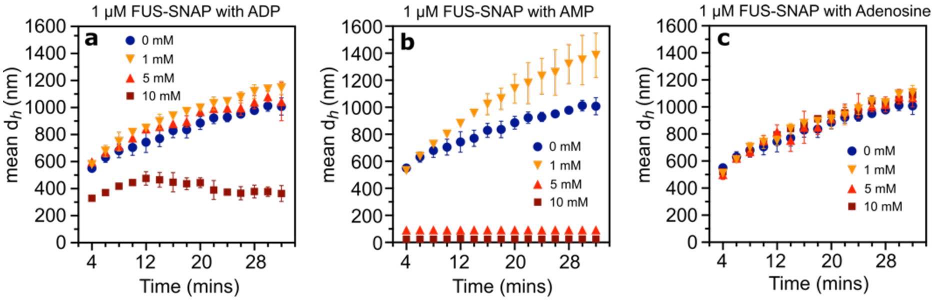

Supplementary figure 7: Adenosine diphosphate (ADP) and adenosine monophosphate (AMP) regulate the cluster size of sub-saturation FUS-SNAP clusters, while adenosine does not. Dynamic Light Scattering data show the hydrodynamic diameter (d_h) of mesoscale clusters at 1 μM FUS-SNAP over 32 minutes with various concentrations of ADP (a), AMP (b), and adenosine (c). Three independent samples (n=3) were used for the measurements, and the data are presented as mean values ± SD.

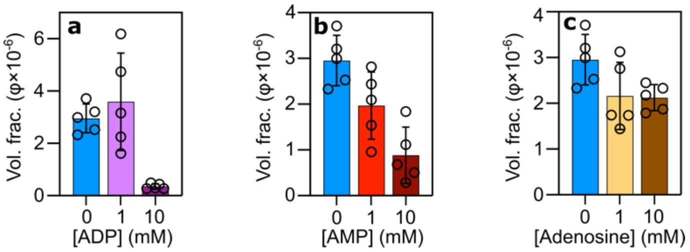

Supplementary figure 8: Modulation of the volume fraction of clusters of FUS-SNAP with Adenosine diphosphate (ADP), adenosine monophosphate (AMP), and adenosine. NTA data for cluster volume fraction at 0.25 μM FUS-SNAP with different ADP (a), AMP (b), and adenosine (c) concentrations. Data are presented as mean ± SD, with n=5 independent samples.

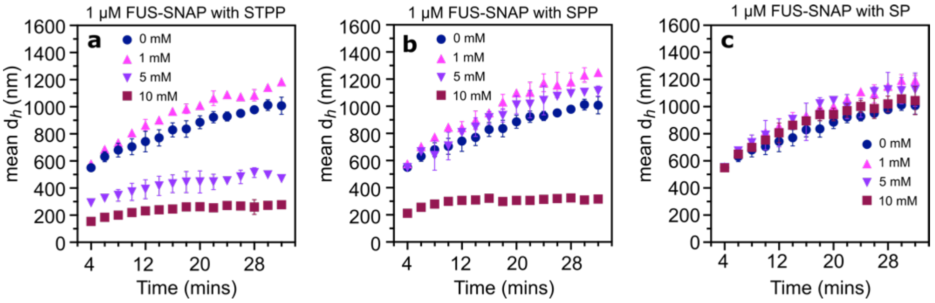

Supplementary figure 9: Phosphates are crucial in regulating the size of sub-saturated FUS-SNAP clusters. Dynamic Light Scattering data show the hydrodynamic diameter (d_h) of mesoscale clusters at 1 μM FUS-SNAP over 32 minutes with varying concentrations of sodium tripolyphosphate (STPP) (a), sodium pyrophosphate (SPP) (b), and sodium phosphate (SP) (c). Three independent samples (n=3) were used for the measurements, and the data are presented as mean values ± SD.

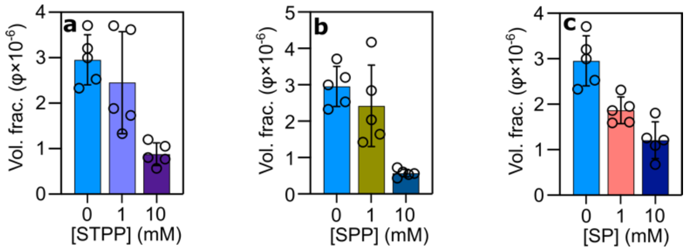

Supplementary figure 10: Modulation of the volume fraction of clusters of FUS-SNAP with sodium tripolyphosphate (STPP), sodium pyrophosphate (SPP), and sodium phosphate (SP). NTA data for cluster volume fraction at 0.25 µM FUS-SNAP with different STPP (a), SPP (b), and SP (c) concentrations. Data are presented as mean ± SD, with n=5 independent samples.

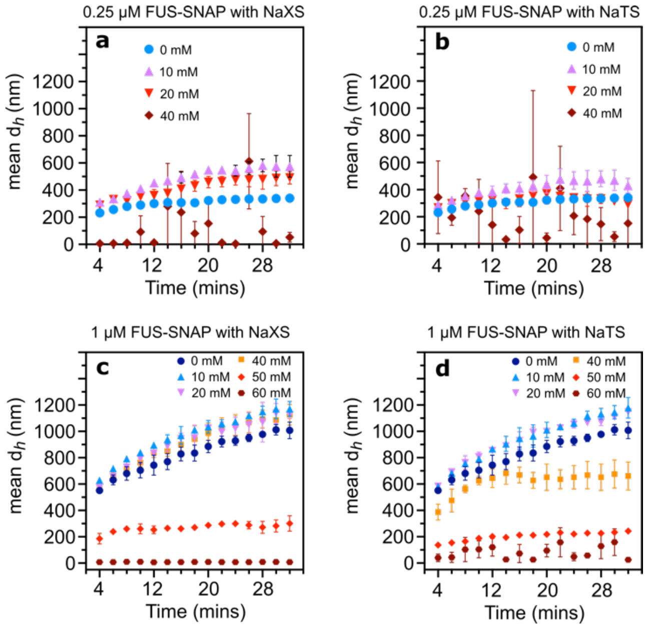

Supplementary figure 11: Hydrotropes modulate the size of saturation clusters. Dynamic light scattering data show the hydrodynamic diameter (d_h) of mesoscale clusters of 0.25 µM FUS-SNAP over 32 minutes with various concentrations of Sodium Xylene Sulfate (NaXS) (a) and Sodium Toluene Sulfate (NaTS) (b). Dynamic light scattering data show the hydrodynamic diameter (d_h) of mesoscale clusters at 1 µM FUS-SNAP over 32 minutes with various concentrations of Sodium Xylene Sulfate (NaXS) (c) and Sodium Toluene Sulfate (NaTS) (d). Three independent samples (n=3) were used for the measurements, and the data are presented as mean values ± SD.

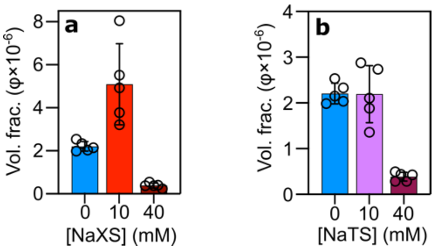

Supplementary figure 12: Modulation of the volume fraction of clusters of FUS-SNAP with sodium xylene sulfonate (NaXS) and sodium toluene sulfonate (NaTS). NTA data for cluster volume fraction at 0.25 µM FUS-SNAP with different NaXS (a), and NaTS (b) concentrations. Data are presented as mean ± SD, with n=5 independent samples.

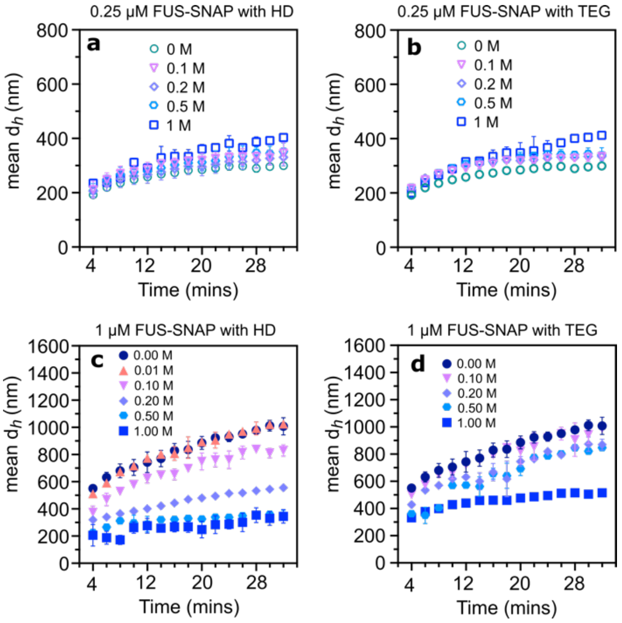

Supplementary figure 13: Hexanediol (HD) and triethylene glycol (TEG) influence the size of condensates differently at different concentrations of FUS-SNAP. Dynamic light scattering data show the hydrodynamic diameter (d_h) of mesoscale clusters of 0.25 µM FUS-SNAP over 32 minutes with different concentrations of HD (a) and TEG (b). Additionally, dynamic light scattering data indicate the hydrodynamic diameter (d_h) of mesoscale clusters at 1 µM FUS-SNAP over 32 minutes with varying concentrations of HD (c) and TEG (d). Three independent samples (n=3) were used for the measurements, and the data are presented as mean values ± SD.

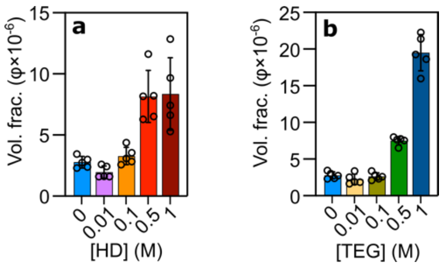

Supplementary figure 14: Modulation of the volume fraction of clusters of FUS-SNAP with Hexanediol (HD), and Triethylene Glycol (TEG). NTA data for cluster volume fraction at 0.25 µM FUS-SNAP with different HD (a), and TEG (b) concentrations. Data are presented as mean ± SD, with n=5 independent samples.

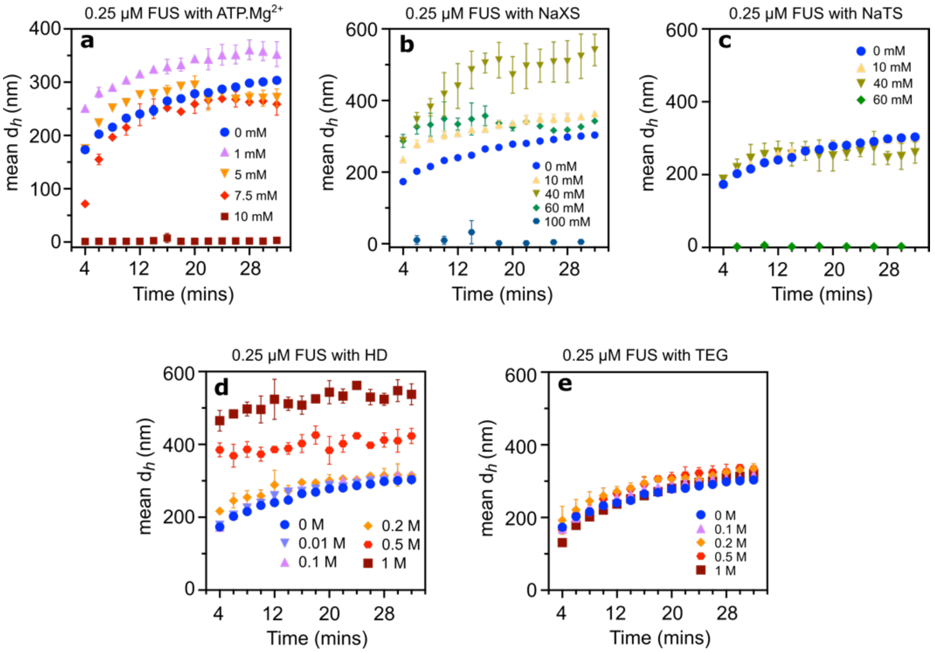

Supplementary figure 15: Sub-saturation cluster formation of untagged FUS is similar to FUS-SNAP in the presence of ATP and hydrotropes but differs with HD and TEG. Dynamic Light Scattering data show mesoscale clusters' hydrodynamic diameter (d_h) at 0.25 µM FUS over 32 minutes with various concentrations of ATP.Mg^{2+} (a), NaXS (b), NaTS (c), HD (d), and TEG (e). Three independent samples (n=3) were used for the measurements, and the data are presented as mean values ± SD.

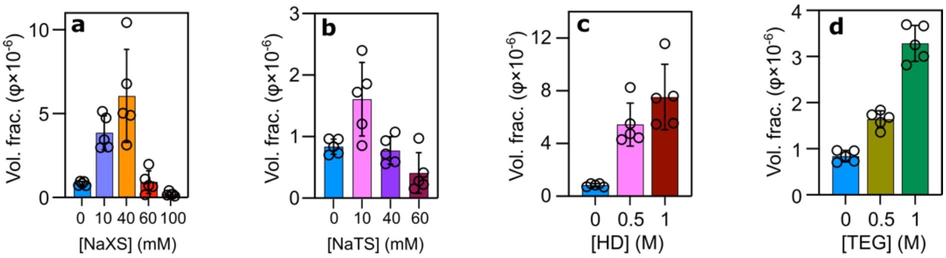

Supplementary figure 16: Modulation of the volume fraction of clusters of FUS with Sodium Xylene Sulfonate (NaXS), Sodium Toluene Sulfonate (NaTS), Hexanediol (HD), and Triethylene Glycol (TEG). NTA data for cluster volume fraction at 0.25 µM FUS with different NaXS (a), NaTS (b), HD (c), and TEG (d) concentrations. Data are presented as mean ± SD, with n=5 (NTA) independent samples.

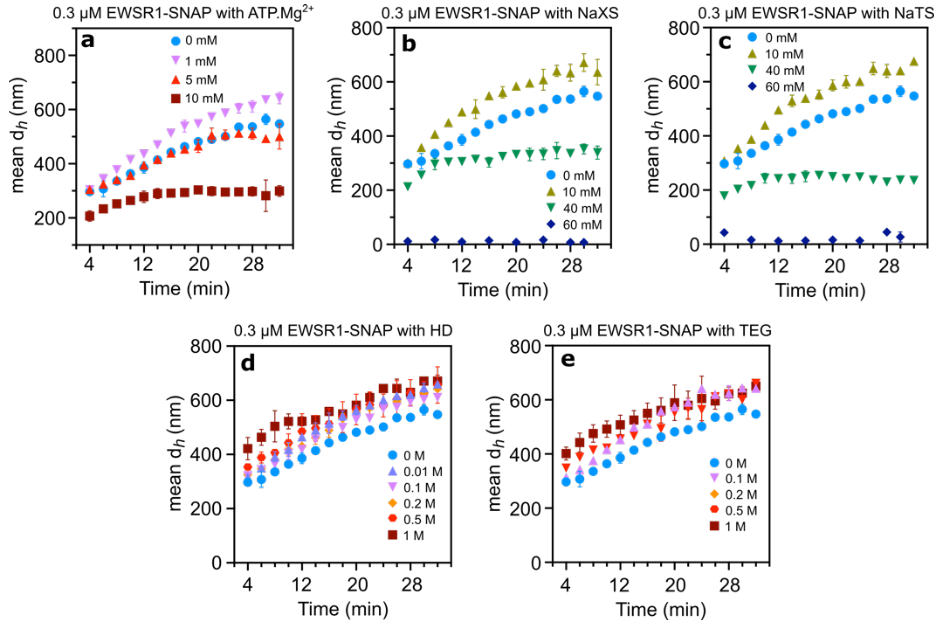

Supplementary figure 17: Sub-saturation cluster formation of EWSR1-SNAP is also influenced by the presence of ATP and hydrotropes but differs with HD and TEG. Dynamic Light Scattering data show mesoscale clusters' hydrodynamic diameter (d_h) at 0.3 µM EWSR1-SNAP over 32 minutes with various concentrations of ATP.Mg^{2+} (a), NaXS (b), NaTS (c), HD (d), and TEG (e). Three independent samples (n=3) were used for the measurements, and the data are presented as mean values ± SD.

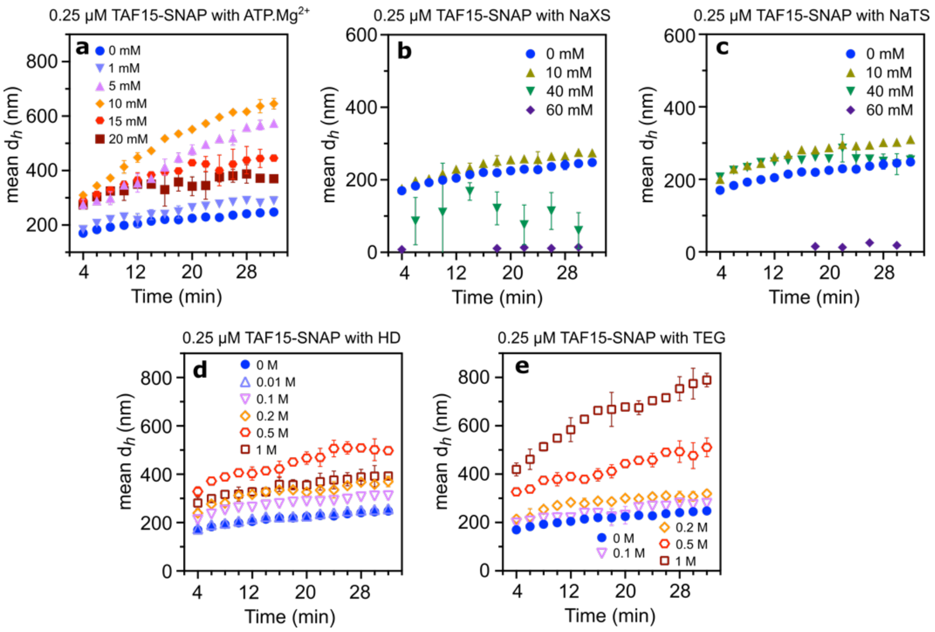

Supplementary figure 18: Sub-saturation cluster formation of TAF15-SNAP is also influenced by the presence of ATP and hydrotropes but differs with HD and TEG. Dynamic Light Scattering data show mesoscale clusters' hydrodynamic diameter (d_h) at 0.25 µM TAF15-SNAP over 32 minutes with various concentrations of ATP.Mg^{2+} (a), NaXS (b), NaTS (c), HD (d), and TEG (e). Three independent samples (n=3) were used for the measurements, and the data are presented as mean values ± SD.

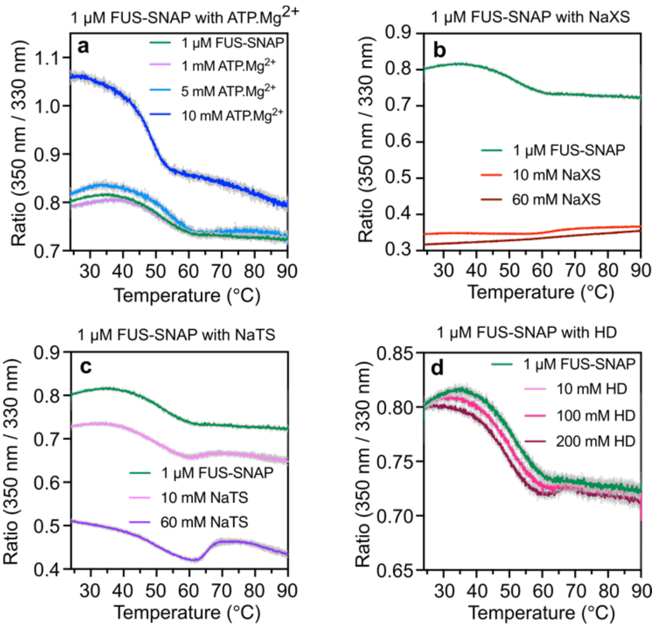

Supplementary figure 19: Interactions with ATP and other small amphiphilic molecules alter the transition temperature of FUS-SNAP probed by NanoDSF. The unfolding curves (350/330) ratio with the apparent transition temperatures of FUS-SNAP in the presence of ATP.Mg^{2+} (a), NaXS (b), NaTS (c), and HD (d). Three independent samples (n=3) were used for the measurements, and the data are presented as mean values ± SD.

Reference

1. Kar M*, et al.* Phase-separating RNA-binding proteins form heterogeneous distributions of clusters in subsaturated solutions. *Proceedings of the National Academy of Sciences* **119**, e2202222119 (2022).
